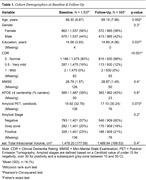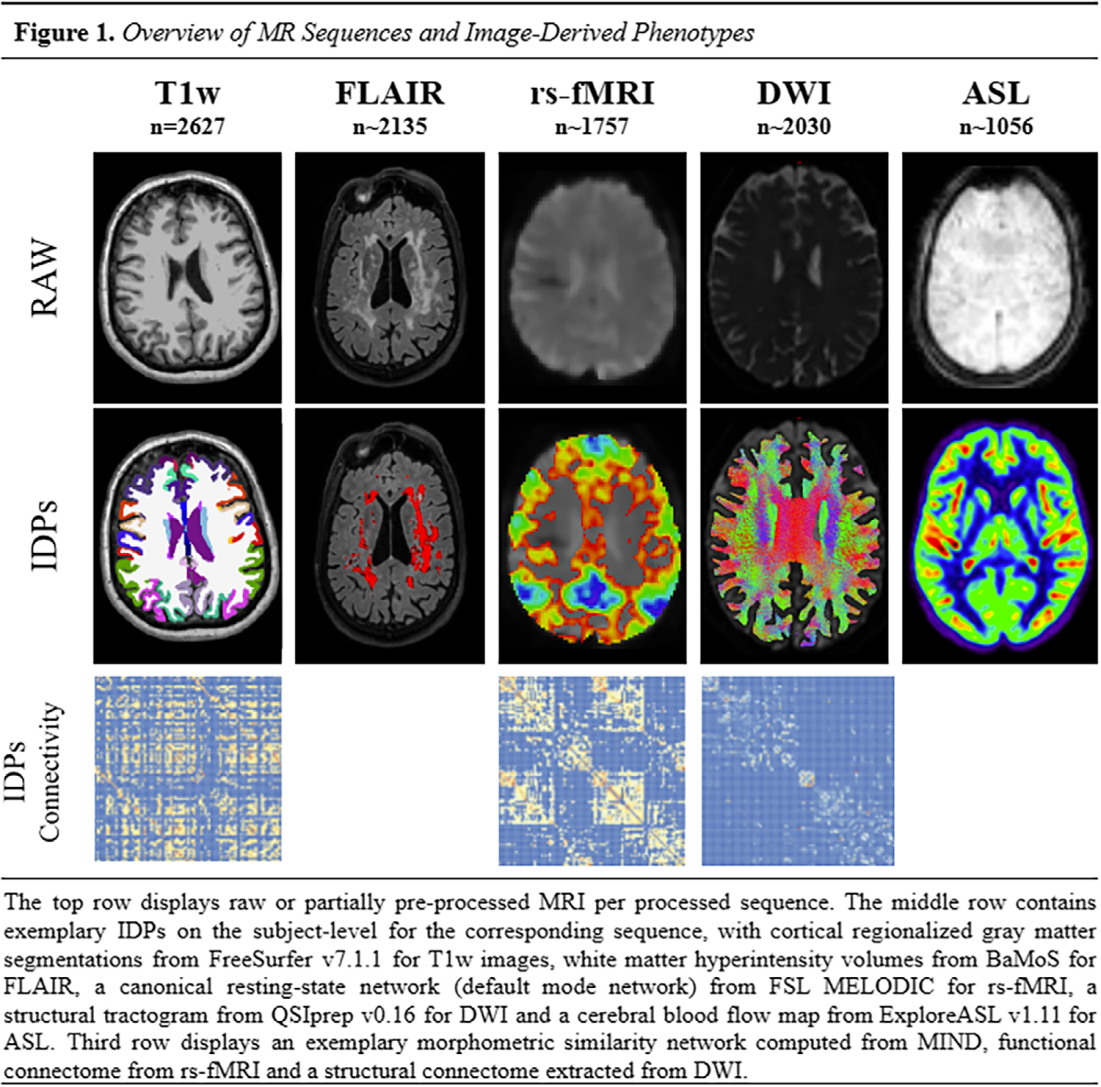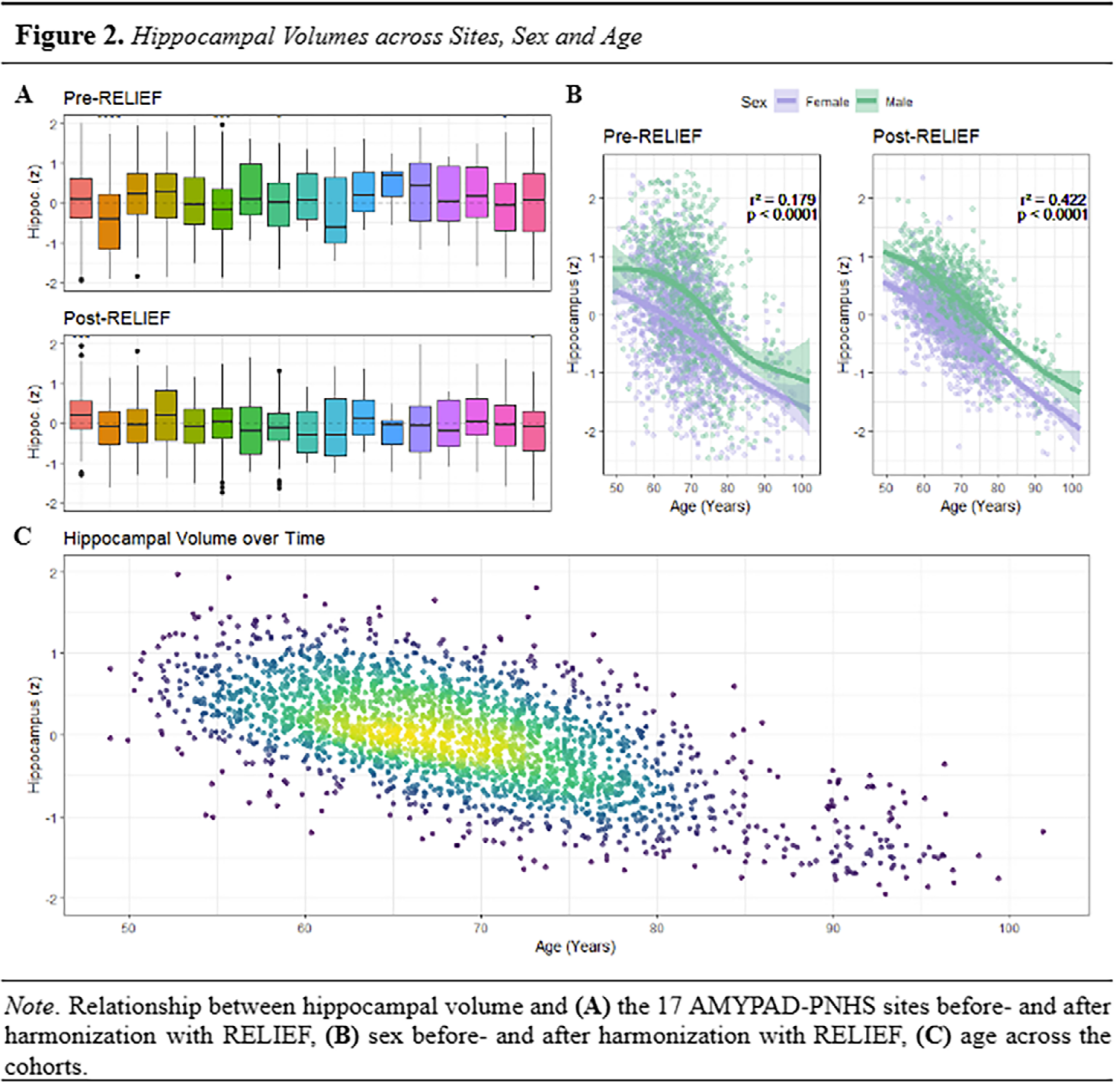# Image‐derived phenotypes in the AMYPAD Prognostic and Natural History Study

**DOI:** 10.1002/alz.093686

**Published:** 2025-01-09

**Authors:** Leonard Pieperhoff, Luigi Lorenzini, Mario Tranfa, Beatriz E Padrela, Craig W Ritchie, Mercè Boada, Marta Marquié, Philip Scheltens, Rik Vandenberghe, Bernard J Hanseeuw, Pablo Martinez‐Lage, Pierre Payoux, Andrew W. Stephens, Christopher Buckley, Gill Farrar, Frank Jessen, Juan Domingo Gispert, David Vállez García, Henk‐Jan Mutsaerts, Lyduine E. Collij, Alle Meije Wink, Frederik Barkhof

**Affiliations:** ^1^ Amsterdam UMC, Amsterdam Netherlands; ^2^ University of Naples Federico II, Naples Italy; ^3^ Centre for Clinical Brain Sciences, The University of Edinburgh, Edinburgh United Kingdom; ^4^ Ace Alzheimer Center Barcelona – International University of Catalunya (UIC), Barcelona Spain; ^5^ University Hospitals Leuven, Leuven Belgium; ^6^ Institute of Neuroscience ‐ UCLouvain, Brussels Belgium; ^7^ Fundación CITA‐Alzhéimer Fundazioa, San Sebastian Spain; ^8^ Université de Toulouse, Toulouse France; ^9^ Life Molecular Imaging GmbH, Berlin Germany; ^10^ GE HealthCare, Amersham United Kingdom; ^11^ University of Cologne, Cologne Germany; ^12^ Barcelona?eta Brain Research Center (BBRC), Barcelona Spain

## Abstract

**Background:**

The Amyloid Imaging to Prevent Alzheimer’s Disease (AMYPAD) Prognostic & Natural History Study (PNHS) is a prospective longitudinal PET cohort of over 1,500 non‐demented individuals from 10 parent cohorts across Europe. We provide an overview of ongoing efforts to curate and integrate magnetic resonance imaging (MRI) multimodal images across sites and to extract biologically meaningful information (i.e., image‐derived phenotypes; IDPs) in this early AD population. Data will be made available on the ADDI platform (fair.addi.ad‐datainitiative.org).

**Method:**

Imaging protocols included core (T1w, T2w, T2*, FLAIR) and advanced (rs‐fMRI, SWI, DWI, and ASL) MRI sequences. Figure 1 provides an overview of the different acquisitions, their corresponding pipelines, and the obtained IDPs. For T1w images, we computed regional volumes and thickness values using FreeSurfer v7.1.1 (surfer.nmr.mgh.harvard.edu/). Morphometric similarity networks were computed using MIND (github.com/isebenius/MIND). White matter hyperintensity volumes were obtained from FLAIR images using Bayesian Model Selection (BaMoS). With rs‐fMRI, mean functional connectivity of canonical networks were extracted using FSL MELODIC and dual‐regression analyses after preprocessing with fMRIPrep v23.0.1 (fmriprep.org). Diffusion MRI scans were processed with QSIprep v0.16 (qsiprep.readthedocs.io) to compute tract‐based spatial statistics and tractograms to build structural connectivity matrices. Finally, cerebral blood flow and the spatial coefficient of variation were computed from ASL images using ExploreASL (github.com/ExploreASL). Statistical harmonization was performed using RELIEF (github.com/junjypark/RELIEF).

**Result:**

Baseline and follow‐up characteristics of the 1537 subjects with available T1w are described in Table 1. Raw and statistically harmonized variants of 380 core and 298 advanced IDPs were calculated. Figure 2 shows the distribution of hippocampal volumes per site before and after statistical harmonization, and their relation with participants' age (R2raw=0.179, R2harmonized=0.422). Considering recent developments in the field, the AMYPAD PNHS aims to include additional IDPs associated with vascular health, such as perivascular spaces and other glymphatic system‐related markers.

**Conclusion:**

We present the pipeline built for MRI harmonization and feature extraction of the AMYPAD PNHS dataset. The extracted IDPs can help identify novel imaging outcomes, support the development of disease progression models for the preclinical stages of AD, and provide a reference point for future studies to promote replicability and robustness of findings.